# Application Value of Intelligent Quick Magnetic Resonance for Accelerating Brain MR Scanning and Improving Image Quality in Acute Ischemic Stroke

**DOI:** 10.2174/0115734056418245250912095159

**Published:** 2025-10-10

**Authors:** Bo Xue, Dengjie Duan, Junbang Feng, Zhenjun Zhao, Jinkun Tan, Jinrui Zhang, Chao Peng, Chang Li, Chuanming Li

**Affiliations:** 1 Department of Radiology, Chongqing University Central Hospital: Chongqing Emergency Medical Center, Chongqing, China; 2 Operations and Technical Support Department of Shanghai Hummingbird Medical Technology Co., Ltd., Shanghai, China; 3 Medic Vision Imaging Solutions Ltd., Haifa, Israel

**Keywords:** Acute ischemic stroke, magnetic resonance imaging, fast imaging, image quality, intelligent quick magnetic resonance

## Abstract

**Introduction:**

This study aimed to evaluate the effectiveness of intelligent quick magnetic resonance (IQMR) for accelerating brain MRI scanning and improving image quality in patients with acute ischemic stroke.

**Methods:**

In this prospective study, 58 patients with acute ischemic stroke underwent head MRI examinations between July 2023 and January 2024, including diffusion-weighted imaging and both conventional and accelerated T1-weighted, T2-weighted, and T2 fluid-attenuated inversion recovery fat-saturated (T2-FLAIR) sequences. Accelerated sequences were processed using IQMR, producing IQMR-T1WI, IQMR-T2WI, and IQMR-T2-FLAIR images. Image quality was assessed qualitatively by two readers using a five-point Likert scale (1 = non-diagnostic to 5 = excellent). Signal-to-noise ratio (SNR) and contrast-to-noise ratio (CNR) of lesions and surrounding tissues were quantitatively measured. The Alberta Stroke Program Early CT Score (ASPECTS) was used to evaluate ischemia severity.

**Results:**

Total scan time was reduced from 5 minutes 9 seconds to 2 minutes 40 seconds, accounting for a reduction of 48.22%. IQMR significantly improved SNR/CNR in accelerated sequences (*P* < .05), achieving parity with routine sequences (P > .05). Qualitative scores for lesion conspicuity and internal display improved post-IQMR (*P* < .05).. ASPECTS showed no significant difference between IQMR and routine images (*P* = 0.79; ICC = 0.91–0.93).

**Discussion:**

IQMR addressed MRI’s slow scanning limitation without hardware modifications, enhancing diagnostic efficiency. The results have been found to align with advancements in deep learning. Limitations included the small sample size and the exclusion of functional sequences.

**Conclusion:**

IQMR could significantly reduce brain MRI scanning time and enhance image quality in patients with acute ischemic stroke.

## 1 INTRODUCTION

Ischemic stroke refers to local brain tissue infarction and necrosis resulting from disrupted blood circulation. Acute ischemic stroke often leads to severe disability, with an average mortality rate of 10% ~ 15% [1]. CT and MRI are the preferred diagnostic methods for acute ischemic stroke. However, low-density lesions typically appear on CT scans after 24 hours. Additionally, CT has limitations in detecting small infarctions, cortical surface infarctions, and posterior fossa infarctions. On the other hand, CT has been found to have certain limitations in pregnant patients [2, 3] and patients receiving long-term radiotherapy [4, 5]. In contrast to CT, MRI offers distinct advantages, including the absence of ionizing radiation and superior soft-tissue resolution. Conventional MRI sequences, such as T1-weighted imaging (T1WI), T2-weighted imaging (T2WI), and T2 fluid-attenuated inversion recovery (T2-FLAIR), have been reported to outperform CT in identifying small infarcts and posterior circulation ischemia [6]. Recent studies have emphasized the importance of early MRI examinations to improve diagnostic accuracy and inform timely treatment decisions for acute ischemic stroke patients [7]. MRI has been reported to exhibit higher sensitivity for detecting early ischemic changes and enable more precise lesion localization [8]. In cases of wake-up stroke (WUS), MRI facilitated estimation of stroke onset time, thereby supporting therapeutic decision-making [9, 10]. However, slow scanning speeds and long examination times have significantly limited MRI use in clinical practice [11].

Currently, several methods exist for shortening MRI scanning times. The simplest approach involves simplifying sequence parameters. However, reducing the number of excitation (NEX) decreases the image SNR, while shortening the repetition time (TR) necessitates balancing SNR and image contrast. Parallel acquisition technology (PAT) uses phased-array coils and algorithms to accelerate imaging by removing image convolution. However, this method provides limited acceleration due to coil receiver component restrictions, negatively affecting SNR and CNR [12, 13]. Simultaneous multi-slice (SMS) technology applies a single radiofrequency pulse to excite multiple layers simultaneously and removes aliasing through reconstruction algorithms. However, SMS technology also reduces SNR and image quality [14, 15]. Compressed sensing (CS) technology employs random sampling and reconstructs signals through nonlinear algorithms, thus reducing redundant data acquisition and shortening scanning times. However, this approach can increase image noise and potentially lose subtle image details during sampling and reconstruction [16-18]. The 3D iterative image reconstruction (IIR) method is a volumetric algorithm used for retrospective reconstruction of MRI images with relatively high noise levels. This method significantly reduces noise, restores image detail, and improves image quality. Multiple 3D IIR technologies exist, such as non-local means, singular value decomposition, sparse representation, machine learning-based techniques, and combinations of these [19-22].

IQMR (Medical Vision Imaging Solutions, Tirat Carmel, Israel) is a post-processing denoising system based on artificial intelligence-assisted IIR technology using parallel processing of multiple graphics processing units. The IQMR algorithm derives from Medic Vision Imaging Solutions’ safe CT algorithm, whose effectiveness in reducing noise and improving image quality in CT images has been validated by previous studies [23, 24]. Previous studies have demonstrated the IQMR technique to effectively reduce noise in brain MRI and facilitate the measurement of brain morphometric parameters [25]. However, its application in cerebrovascular diseases has not yet been reported. The present study aimed to evaluate the effectiveness of IQMR in accelerating brain MRI scanning speed and improving image quality for patients with acute ischemic stroke.

## 2 MATERIALS AND METHODS

### 2.1 Subjects

This study was approved by the ethics committee of our institution (EC review no. (36) of 2023). Written informed consent was obtained from each patient. Patients with acute ischemic stroke were consecutively enrolled at our institution from July 2023 to January 2024.

The inclusion criteria were as follows: (1) first-time presentation of cerebrovascular disease symptoms; (2) focal neurological deficits (*e.g*., weakness or numbness of one side of the face or limbs, language disorders); (3) age range of 18–80 years; (4) diagnosis of acute ischemic stroke confirmed by DWI; and (5) interval between symptom onset and MRI examination less than 24 hours.

The exclusion criteria were as follows: patients with general contraindications to MRI, other neurological disorders, severe claustrophobia, inability to complete the examination, or severe image artifacts. The flow chart of the study design is shown in Fig. (**1**).

### 2.2 MR Acquisition

All patients underwent head MRI using a 1.5T MR system (uMR560, United Imaging Healthcare, China) with a 16-channel head coil. The diffusion-weighted imaging sequence was performed with a TR of 3576 msec, echo time (TE) of 97.6 msec, voxel size of 1.88×1.88×5 mm^3^, and bandwidth of 1480 kHz/pixel.

Routine sequences (T1-weighted, T2-weighted, and T2-FLAIR) were acquired with the following parameters: (1) T1-weighted turbo spin echo sequence: TR = 532 msec; TE = 10.84 msec; voxel size = 1.56×0.94×5 mm^3^; bandwidth = 150 kHz/pixel; echo train length = 3; average = 3; (2) T2-weighted turbo spin echo sequence: TR = 4569 msec; TE = 95.7 msec; voxel size = 1.56×0.94×5 mm^3^; bandwidth = 250 kHz/pixel; echo train length = 20; average = 3; (3) T2-FLAIR sequence: TR = 7000 msec; TE = 75.88 msec; voxel size = 1.56×0.94×5 mm^3^; bandwidth = 150 kHz/pixel; echo train length = 16; average = 2. Finally, accelerated sequences (T1WI, T2WI, and T2-FLAIR) were additionally employed with average reduced to 1; other parameters remained unchanged.

### 2.3 IQMR Processing

IQMR (Medic Vision Imaging Solutions, Tirat Carmel, Israel) combines deep learning with traditional image enhancement technologies to improve image quality and enable shorter MRI scan times, encompassing the following: iterative image reconstruction (IIR), edge enhancement, super-resolution, k-space correction, and image personalization.

Initially, the input image is processed using a convolutional neural network (CNN) to increase the visibility of small image details and improve the image sharpness. The input images are enhanced through a cascade of filter banks. Thresholding and scaling operations are applied during this process. The system optimizes filter parameters through an image-guided process, where image couples of high- and low-resolution images are used to optimize the filter weights. The CNN architecture is based on a modified version of SRGAN with modified filter blocks and loss functions. The algorithms are trained to produce higher-resolution and sharper images from their low-resolution counterparts. Training was done using a large set of MRI datasets (over 500,000) obtained from scanners of various vendors and clinical sites with a variety of clinical indications and field strengths, thus experiencing a wide range of image quality, tissue contrasts, acquisition parameters, and patient anatomies.

The input image was rescaled to match the dimensions of the acquired scanner's k-space data, prior to zero-filling (a technique widely used to interpolate the image by increasing the matrix size of the acquired raw data prior to Fourier transforming the k-space data). This allowed the following iQMR processing stages to be applied on a more accurate representation of the acquired signal and noise characteristics, and improved the overall performance of the processing.

IQMR’s iterative image reconstruction (IIR) is a volumetric algorithm for retrospective reconstruction of MRI scans with relatively high noise levels, significantly reducing noise, restoring the fine details underneath the noise, and improving certain image quality parameters. The input data set (MRI images) is decomposed into 3D patches, which are transformed into feature space by calculating multiple features for each image patch. These patches are then grouped based on a unique similarity measure. By combining knowledge of the similarity between patches and the noise statistics estimate, the noise and signal are jointly estimated and separated. This process is repeated until specific convergence criteria are met.

iQMR edge enhancement algorithms enhance edge contrast to improve its apparent (or perceived) sharpness by identifying sharp edge boundaries and increasing the image contrast in the area immediately around the edge, without affecting details in more uniform areas of the image.

Denoised images may appear unnatural to radiologists, who are accustomed to interpreting MRI scans that contain a certain amount of noise, even when the images are acquired under optimal conditions. To address this, a weighted average of the processed and original images, combined with user-selected preferences (soft, sharp,* etc.*), is used.

Following all these image enhancement steps, the processed image is converted and rescaled back to the original reconstructed image dimensions, along the slice axis and the required slice thickness.

The entire processing sequence took approximately 20 seconds; all images were stored and analyzed in DICOM format.

### 2.4 Qualitative Image Analysis

Qualitative analysis was performed independently by two readers (with 10 years of brain MRI experience) using the IMPAX Client workstation (version 6.5.3.3009 2015, AGFA HealthCare, Belgium). Both readers were blinded to clinical information and sequence types. Images from routine, accelerated, and IQMR sequences were evaluated for artifacts (motion, ringing, partial volume, and susceptibility artifacts), lesion conspicuity (contrast between lesion and surrounding tissue), lesion internal display (detailed lesion structures), and overall image quality (general image impression). A five-point Likert scale was used to rate each category, and scores obtained by both readers were averaged. Scoring criteria are provided in Appendix Table **1**.

### 2.5 Quantitative Image Analysis

SNR and CNR values of lesions and surrounding tissues were calculated and analyzed for routine, accelerated, and IQMR sequences. Regions of interest (ROIs) measuring 20 mm^2^ were drawn on lesions and corresponding surrounding tissues. The average signal intensity within the ROI represented regional signal values, while the standard deviation (SD) of signal intensity within the ROI represented regional noise. SNR was calculated by dividing the ROI signal intensity by the SD. CNR was calculated by dividing the difference in signal intensity between lesions and surrounding areas by the mean SD value. Weighted kappa tests were used to assess consistency between the reviewers.

### 2.6 ASPECTS Assessment

Severity of cerebral infarction was evaluated using the ASPECTS [26, 27]. ASPECTS is a simple, reliable, and systematic scoring system that allows quick, semi-quantitative evaluation, aiding physicians in predicting thrombolytic effectiveness and long-term patient prognosis. Two radiologists, blinded to clinical data, independently assessed routine and IQMR images and assigned corresponding scores. Final scores represented the average of the two assessments.

### 2.7 Statistical Analysis

Statistical analyses were conducted using SPSS software (version 25.0). The Shapiro-Wilk test was performed to assess data normality. Normally distributed data were compared among groups using one-way ANOVA and the post-hoc Dunnett-t test. Non-normally distributed data were analyzed using the Friedman test and post-hoc Bonferroni test. Weighted kappa values were interpreted as follows: ≥0.81 indicated almost perfect agreement; 0.61-0.80, substantial agreement; 0.41-0.60, moderate agreement; 0.21-0.40, fair agreement; and 0.00-0.20, slight agreement. Overall consistency of ASPECTS scores between readers was assessed by calculating two-way mixed absolute consistency intraclass correlation coefficients (ICC). Bonferroni correction was used for P-value adjustments, and *P* <0.05 indicated statistical significance.

## 3 RESULTS

A total of 58 patients (31 males, 27 females; mean age, 63±12 years; range, 29–80 years) were included. The clinical characteristics of the enrolled participants are presented in Table **1**. The total acquisition time for routine T1WI, T2WI, and T2-FLAIR sequences was 5 minutes and 9 seconds. The total examination time for IQMR-accelerated sequences was 2 minutes and 40 seconds, representing a 48.22% reduction. Examination times for T1WI, T2WI, and T2-FLAIR sequences were reduced by 55.84%, 52.17%, and 42.95%, respectively. Fifty-eight lesions were identified, with no difference observed among conventional, accelerated, and IQMR images.

### 3.1 Comparison of Qualitative Image Evaluations

**Fig. (2):**
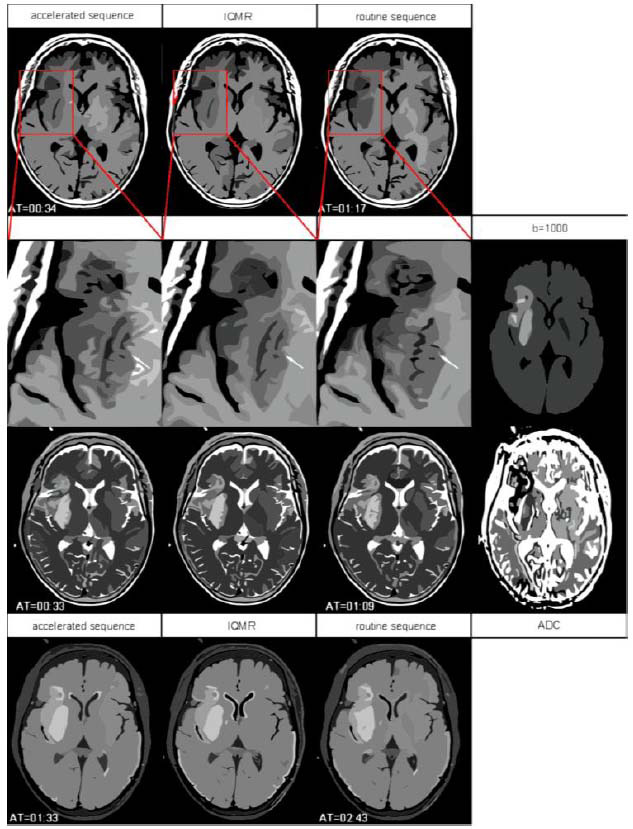
Acute ischemic stroke in a 67-year-old female patient. Compared to routine sequences, accelerated sequences shortened examination times by 55.84% (T1WI), 52.17% (T2WI), and 42.95% (T2-FLAIR). However, accelerated sequences showed increased image noise and lower overall quality. After IQMR post-processing, lesion conspicuity, internal lesion detail, and overall image quality significantly improved. IQMR images provided better internal lesion display (white arrow) and equivalent lesion conspicuity and overall image quality compared to routine sequences.

Statistical analysis showed accelerated sequences to be inferior to routine sequences in terms of lesion conspicuity and overall image quality, but superior in terms of artifacts (*P* < 0.05). After IQMR processing, significant improvements occurred in lesion conspicuity, lesion internal display, and overall image quality (*P* < 0.05) (Fig. **2**). However, IQMR processing did not further improve the artifacts (*P* ≥ 0.05). Compared to routine sequences, IQMR-T1WI, IQMR-T2WI, and IQMR-T2-FLAIR images showed significantly reduced artifacts, improved lesion internal display, and equivalent lesion conspicuity and overall image quality (*P* ≥ 0.05) (Table **2**). Intra-reader reliability was strong (ICC: 0.76–0.93).

### 3.2 Comparison of Quantitative Image Evaluations

**Fig. (3):**
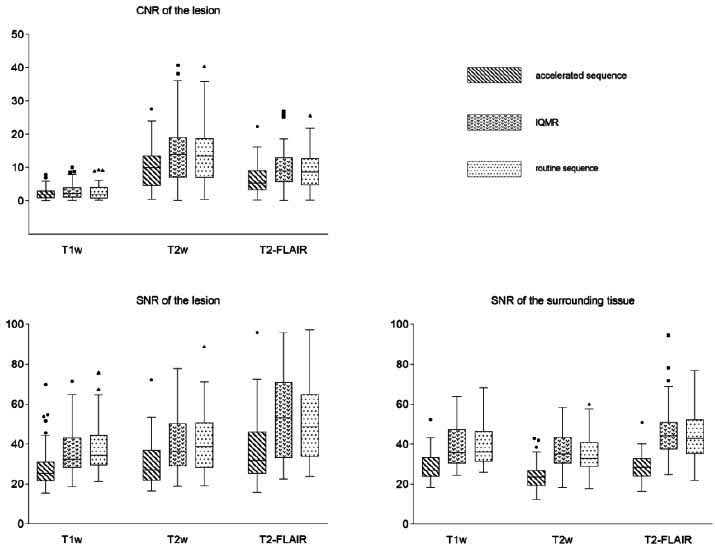
SNR and CNR of routine, accelerated, and IQMR sequences. After IQMR processing, the SNR and CNR of accelerated T1WI, T2WI, and T2-FLAIR sequences significantly improved (*P* < 0.05). No statistically significant differences were observed in SNR and CNR of lesions and surrounding tissues between IQMR and routine sequences (*P* ≥ 0.05).

Statistical analysis indicated that after IQMR processing, the SNR and CNR of lesions and surrounding tissues in accelerated T1WI, T2WI, and T2-FLAIR images significantly improved (*P* < 0.05). No statistically significant difference appeared between IQMR-processed and routine images (*P* ≥ 0.05) (Fig. **3**).

### 3.3 ASPECTS Assessment

No statistically significant difference in ASPECTS scores occurred between IQMR and routine sequences (*P* = 0.79). The intra-reader reliability was strong, with an ICC of 0.91 and 0.93.

## 4 DISCUSSION

Acute ischemic stroke is a common neurological condition in clinical practice. Rapid diagnosis and treatment are crucial for reducing disability and mortality rates [28, 29]. MRI remains the most critical method for detecting and assessing ischemic stroke; however, its slow scanning speed remains a significant challenge. Accelerating scan speed and shortening examination duration can facilitate timely treatment and improve patient outcomes. For patients with impaired consciousness unable to remain stationary for prolonged periods, shorter scan times can significantly improve MRI examination success rates. Additionally, faster examinations can reduce motion artifacts and enhance clinical efficiency. In this study, accelerated sequences were obtained by reducing the average values from conventional sequences, which consequently reduced image SNR. The resulting images underwent IQMR processing on an external platform for noise reduction, sharpening, and enhancement. IQMR divided raw data into small 3D modules, separated signals and noise, and iteratively optimized the image until achieving ideal quality standards. IQMR images demonstrated excellent overall image quality, lesion conspicuity, and lesion internal structure display. Clinically, high-quality images and clear internal lesion detail are essential for assessing the timing and severity of cerebral infarction. Results indicated that IQMR improved lesion evaluation and had substantial clinical value. Compared to routine T1WI, T2WI, and T2-FLAIR sequences, examination times were reduced by 55.84%, 52.17%, and 42.95%, respectively. This considerable reduction in examination time enabled earlier diagnosis and significant clinical utility in cerebral infarction. For patients with early cerebral infarction, brain density changes on CT were subtle, with reported average sensitivities of 20%-87% and specificities of 56%-100% [30]. MRI examinations are more sensitive to changes in free water content within infarcted tissues. In this study, all cerebral infarction lesions were detected using accelerated IQMR images, and the ASPECT scores were consistent with routine sequences. This indicated that IQMR technology could accurately assess lesion extent and severity in acute ischemic stroke.

SNR and CNR are essential indicators of MRI image quality. SNR is the ratio between signal intensity and background noise, reflecting image clarity. CNR is the ratio of signal intensity difference between tissues and background noise, indicating image contrast. This study demonstrated that after IQMR post-processing, the SNR and CNR of T1WI, T2WI, and T2-FLAIR images significantly improved, showing no significant differences compared to traditional sequences for lesions and surrounding tissues. This improvement is clinically valuable, as it enhanced lesion detection and reduced missed diagnoses.

In previous studies, Evan *et al.* [31] developed a deep-learning algorithm to reconstruct high-speed, two-dimensional cine cardiac MRI sequences. They found that although cardiac MRI acquisition time was shortened compared to standard balanced steady-state free precession, image quality decreased. Maennlin *et al.* [32] compared image quality, noise, artifacts, and diagnostic confidence of deep-learning-based volumetric interpolated breath-hold examination (VIBE) sequences with standard VIBE sequences in 50 chest MR examinations. Compared to these studies, this study was the first to utilize artificial intelligence in investigating rapid MRI examination technology for stroke patients who require faster diagnosis and treatment. Our method could significantly reduce the total examination time, while improving internal lesion visualization and enhancing overall image quality, thus demonstrating advantages over the previously reported methods.

The IQMR used in this study is a post-processing algorithm that does not alter MRI software, hardware, or examination procedures. It can process images obtained by MRI scanners from any manufacturer, enhancing its applicability. Currently, many MRI systems worldwide, particularly in developing countries, operate at or below 1.5T field strength. This technology can significantly improve image quality and scanning speed without additional hardware costs, which is clinically beneficial. Kanewska *et al.* [33] found that deep-learning algorithms significantly accelerated scanning speed and reduced noise in shoulder joint MRI. Bischoff *et al.* [34] employed deep-learning reconstruction combined with CS to enable rapid prostate cancer imaging. However, these techniques require specialized hardware and cannot be universally implemented on existing MRI scanners. Compared to these methods, the IQMR algorithm used in this study does not require advanced equipment and is compatible with MR systems from any manufacturer.

## 5 STUDY LIMITATIONS

This study involved several limitations. First, only DWI, T1WI, T2WI, and T2-FLAIR sequences were included, and specialized functional sequences, such as SWI and PWI, were not assessed. Second, the sample size was small, and dynamic tracking was not conducted. Third, only images from a 1.5T MRI system were analyzed, excluding lower-field-strength images. Future studies should include larger sample sizes, additional sequences, dynamic follow-up, and evaluation of MRI systems at different field strengths to confirm the clinical utility of this technology.

## CONCLUSION

In summary, IQMR significantly accelerated MRI acquisition and reduced scanning time, addressing a key clinical limitation of conventional MRI. IQMR-derived images demonstrated high diagnostic accuracy for acute ischemic stroke, providing superior image quality with enhanced lesion conspicuity and detailed visualization of infarct internal structures. These findings supported the clinical utility of IQMR for early diagnosis and management of acute ischemic stroke.

**Appendix 1:** Qualitative analysis of subjective scoring criteria.

-	5	4	3	2	1
Lesion internal display	Sharp edges and clearly identifiable internal structure	Sharper boundary, clearer internal structure	The boundary is blurred, and the internal structure can still be distinguished	The outline is obviously distorted, and the internal structure is slightly visible	Boundary contours and internal structures are almost unrecognizable
Artifacts	Artifact free	Mild artifact	Moderate artifacts	Obvious artifact	Severe artifacts
Lesion conspicuity	Lesions >3 mm and ≤3 mm are clearly visible	Lesions>3 mm clearly visible, lesions ≤3 mm visible	Lesions >3 mm visible, lesions ≤3 mm slightly visible	Lesions >3 mm are slightly visible, lesions ≤3 mm are not visible	Lesions >3 mm and ≤3 mm are not visible
Overall image quality	Excellent	Good	Moderate	Bad	Non-diagnostic

## Figures and Tables

**Fig. (1):**
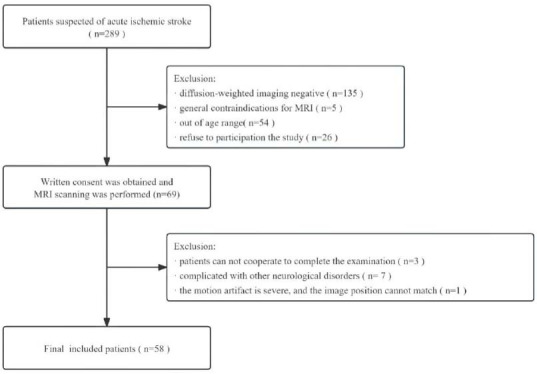
Flow diagram of patient selection.

**Table 1:** Clinical characteristics of enrolled participants.

**Variable**	**Value**
No. of participants	58
Male/Female	31/27
Age (y)*	63±12
Time from stroke onset (last known well) to MRI sequence (h)	11.3±4.5
Causes of cerebral infarction	
Large-artery atherosclerosis	21
Cardioembolism	19
Small-vessel occlusion	13
Acute stroke of other determined etiology	3
Stroke of undetermined etiology	2
ASPECTS score	
10	4 (4)
9	20 (20)
8	14 (11)
7	6(6)
6	5(6)
5	1(1)
4	1(1)
3	4(4)
2	2(2)
1	0(0)
0	1(1)

**Note:** Except where indicated, data present the numbers of patients. * Data have been expressed as mean ± SD. TOAST = Trial of Org 10172 in Acute Stroke Treatment, ASPECTS = Alberta Stroke Program Early CT Score.

**Table 2:** Comparison of qualitative image evaluations.

**Category**		**Routine sequences**	**Accelerated sequences**	**IQMR**	**Friedman *P*-value**
Artifacts	T1w	4.00 (3.00, 4.00) ^(1)^ ^(2)^	4.00 (4.00, 5.00) ^(3)^	4.00 (4.00, 5.00) ^(3)^	< 0.001
	T2w	4.00 (4.00, 4.00) ^(1)^ ^(2)^	4.00 (4.00, 5.00)^(3)^	4.00 (4.00, 5.00)^(3)^	< 0.001
	T2-FLAIR	4.00 (3.50, 4.50)^(1)^ ^(2)^	4.00 (4.00, 5.00)^(3)^	4.50 (4.00, 5.00)^(3)^	< 0.001
Lesion internal display	T1w	3.00 (2.88, 3.00) ^(2)^	3.00 (3.00, 3.00) ^(2)^	3.00 (3.00, 4.00)^(1)^^(3)^	< 0.001
	T2w	3.00 (3.00, 4.00) ^(2)^	3.00 (3.00, 4.00) ^(2)^	4.00 (4.00, 4.00)^(1)^^(3)^	< 0.001
	T2-FLAIR	3.00 (3.00, 3.63) ^(2)^	3.00 (3.00, 4.00) ^(2)^	4.00 (3.88, 4.00)^(1)^^(3)^	< 0.001
Lesion conspicuity	T1w	4.00 (3.00, 4.00) ^(1)^	3.00 (2.50, 3.00) ^(2)^ ^(3)^	4.00 (3.00, 4.00)^(1)^	< 0.001
	T2w	4.00 (4.00, 4.00) ^(1)^	3.50 (3.00, 4.00) ^(2)^^(3)^	4.00 (3.50, 4.50)^(1)^	< 0.001
	T2-FLAIR	4.00 (3.00, 4.00)^(1)^	3.00 (3.00, 4.00) ^(2)^ ^(3)^	4.00 (3.00, 4.00)^(1)^	< 0.001
Overall image quality	T1w	4.00 (3.00, 4.00)^(1)^	3.00 (3.00, 3.00) ^(2)^ ^(3)^	4.00 (3.00, 4.00) ^(1)^	< 0.001
	T2w	4.00 (4.00, 5.00) ^(1)^	3.50 (3.50, 4.00) ^(2)^ ^(3)^	4.00 (4.00, 5.00) ^(1)^	< 0.001
	T2-FLAIR	4.00 (4.00, 5.00) ^(1)^	3.00 (3.00, 4.00) ^(2)^ ^(3)^	4.00 (4.00, 5.00)^(1)^	< 0.001

**Notes:** Data are medians, with interquartile ranges in parentheses. A five-point Likert scale was used for each category (1 = nondiagnostic, 5 = excellent). T1w = T1-weighted turbo spin echo (TSE) sequence; T2w = T2-weighted TSE sequence; T2-FLAIR = T2 fluid-attenuated inversion recovery fat-saturated sequence. ^(1)^ Post hoc Bonferroni test, *P* < 0.05 versus accelerated sequences. ^(2)^ Post hoc Bonferroni test, *P* < 0.05 versus iQMR-accelerated sequences. ^(3)^ Post hoc Bonferroni test, *P* < 0.05 versus routine sequences.

## Data Availability

All data generated or analyzed during this study are included in this published article.

## LIST OF ABBREVIATIONS

IQMR: Intelligent quick magnetic resonance
FLAIR: Fluid-attenuated inversion recovery
FS: Fat saturated
NEX: Number of excitations
TR: Repetition time
PAT: Parallel acquisition technology
SNR: Signal-to-noise ratio
CNR: Contrast-to-noise ratio
SMS: Simultaneous multi-slice
CS: Compressed sensing
IIR: Iterative image reconstruction
DWI: Diffusion weighted imaging
CNN: Convolutional neural network
ASPECTS: Alberta Stroke Program Early CT Score
DL: Deep learning
ROI: Region of interest
SD: Standard deviation
DICOM: Digital imaging and communications in medicine
ICC: Intraclass correlation coefficient
VIBE: Volumetric interpolated breath-hold examination
SWI: Susceptibility weighted imaging
PWI: Perfusion weighted imaging

## STANDARDS OF REPORTING

STROBE guidelines were followed.
